# Supplementary material for: Cryo-EM structures of the TTYH family reveal a novel architecture for lipid interactions
Source: Nat Commun. 2021 Aug 12;12:4893. doi: 10.1038/s41467-021-25106-4 (PMC8361169; doi:10.1038/s41467-021-25106-4)
Supplement: Supplementary file 1 — Supplementary Information [file 41467_2021_25106_MOESM1_ESM.pdf]

Supplementary Information

Cryo-EM structures of the TTYH family reveal a novel architecture for lipid interactions

Anastasiia Sukalskaia, Monique S. Straub, Dawid Deneka, Marta Sawicka and Raimund Dutzler

Supplementary Figures

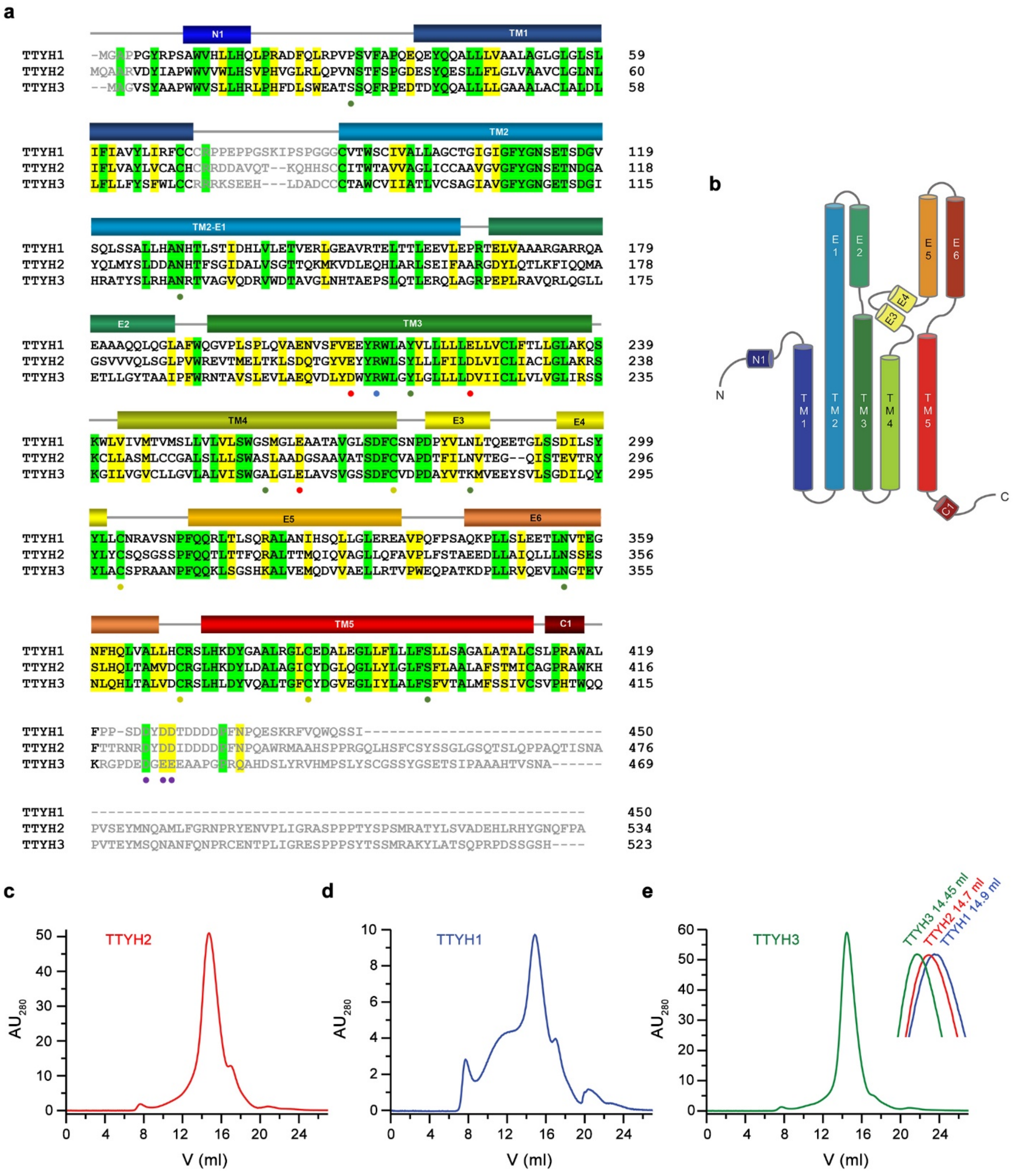

Supplementary Fig. 1: Sequence, topology and biochemical properties. a Sequence alignment of human paralogs TTYH1 (NCBI: NP_116035), TTYH2 (NCBI: NP_116035) and TTYH3 (GenBank: AAI31825.1). Identical residues are highlighted in green, homologous residues

in yellow. Secondary structure of TTYH2 is shown above. Selected residues are labeled (●): blue, basic, green, polar and red, acidic residues in transmembrane domain, yellow, cysteines involved in disulfide bridges, violet, residues of an acidic cluster at the disordered C-terminus predicted to be involved in Ca^{2+}-binding. **b** Schematic topology of the TTYH subunit. Colors are as in **a**. **c–e** Size-exclusion profiles of TTYH proteins purified for structure determination, separated on a Superose 6 column. **c** TTYH2, **d** TTYH1, **e** TTYH3 with inset showing a comparison of the normalized peaks of different paralogs.

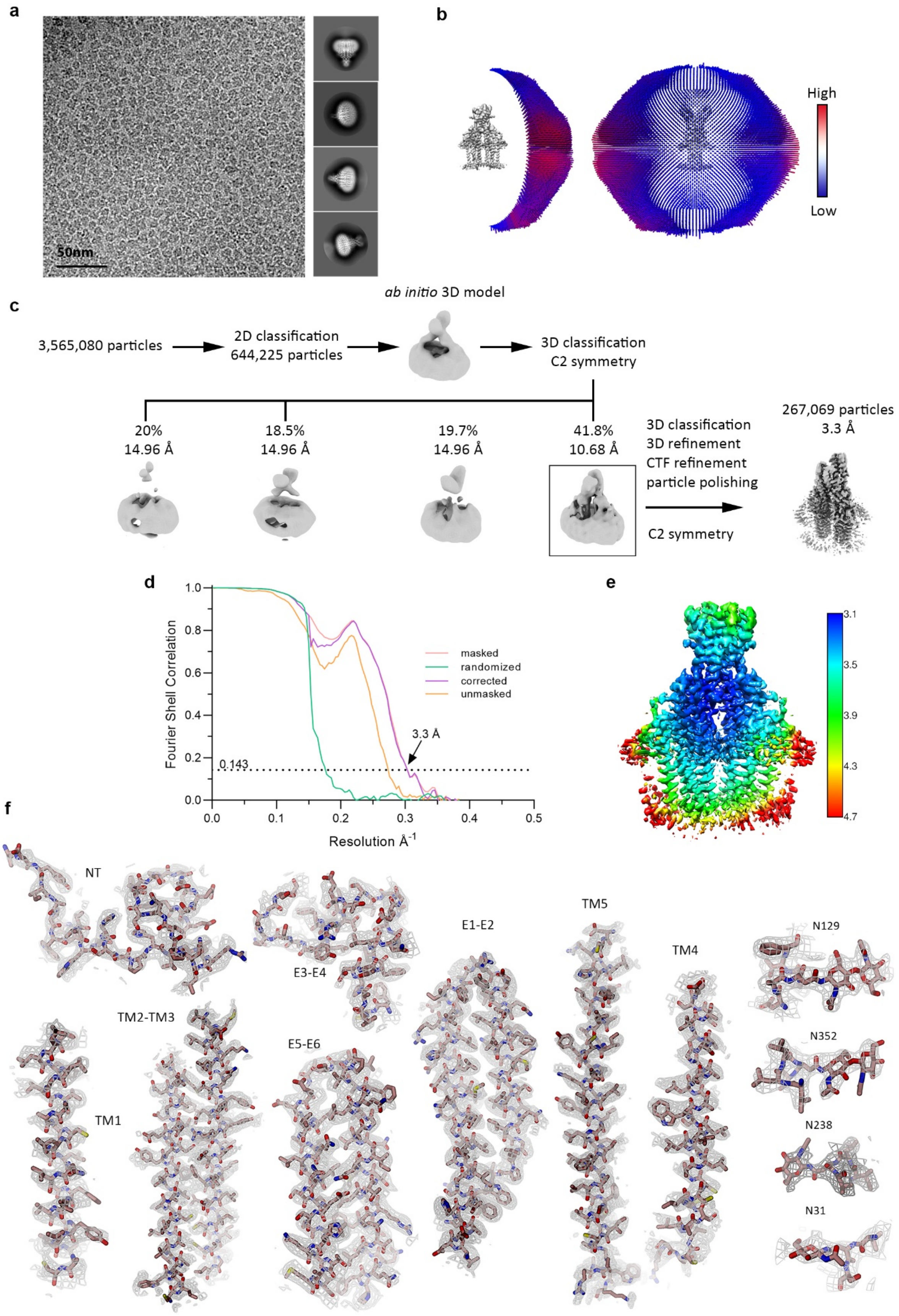

a
50nm
b
High
Low
c
ab initio 3D model
3,565,080 particles
2D classification
644,225 particles
3D classification
C2 symmetry
20%
14.96 Å
18.5%
14.96 Å
19.7%
14.96 Å
41.8%
10.68 Å
3D classification
3D refinement
CTF refinement
particle polishing
C2 symmetry
267,069 particles
3.3 Å
d
Fourier Shell Correlation
Resolution Å-1
masked
randomized
corrected
unmasked
0.143
3.3 Å
e
3.1
3.5
3.9
4.3
4.7
f
NT
E3-E4
E1-E2
TM5
TM4
N129
N352
N238
N31
TM1
TM2-TM3
E5-E6

Supplementary Fig. 2: Cryo-EM structure of TTYH2 in GDN. a Representative cryo-EM micrograph (of a total of 5,468) and 2D class averages of TTYH2. **b,** Angular distribution of particles used for the final 3D reconstruction of TTYH2. The length and the color of cylinders correspond to the number of particles with respective Euler angles. **c,** Data processing workflow. Initially picked 3,565,080 particles were subjected to multiple rounds of 2D classification resulting in a particle set used for generation of an *ab initio* model. The reconstruction showed clear C2 symmetry features and was used as a reference in a subsequent 3D classification. The distribution of all particles (%) and the resolution of each 3D class are indicated. Particles from the best-looking 3D class displaying clearly resolved features of the protein were used for the map refinement resulting in a 3.3 Å final map of TTYH2. **d** FSC plots of the final TTYH2 reconstruction: unmasked (yellow), masked (pink), randomized (green) and corrected for mask convolution effects (purple). The resolution at which the FSC curve of the map, which was corrected for mask convolution effects drops below 0.143 is indicated. **e** Final 3D reconstruction of TTYH2 coloured according to local resolution. **f** Sections of the cryo-EM density (grey mesh, contoured at 5.5 σ) superimposed on the model. The different regions of the protein are labelled. N31, N129, N238 and N352 correspond to glycosylated residues.

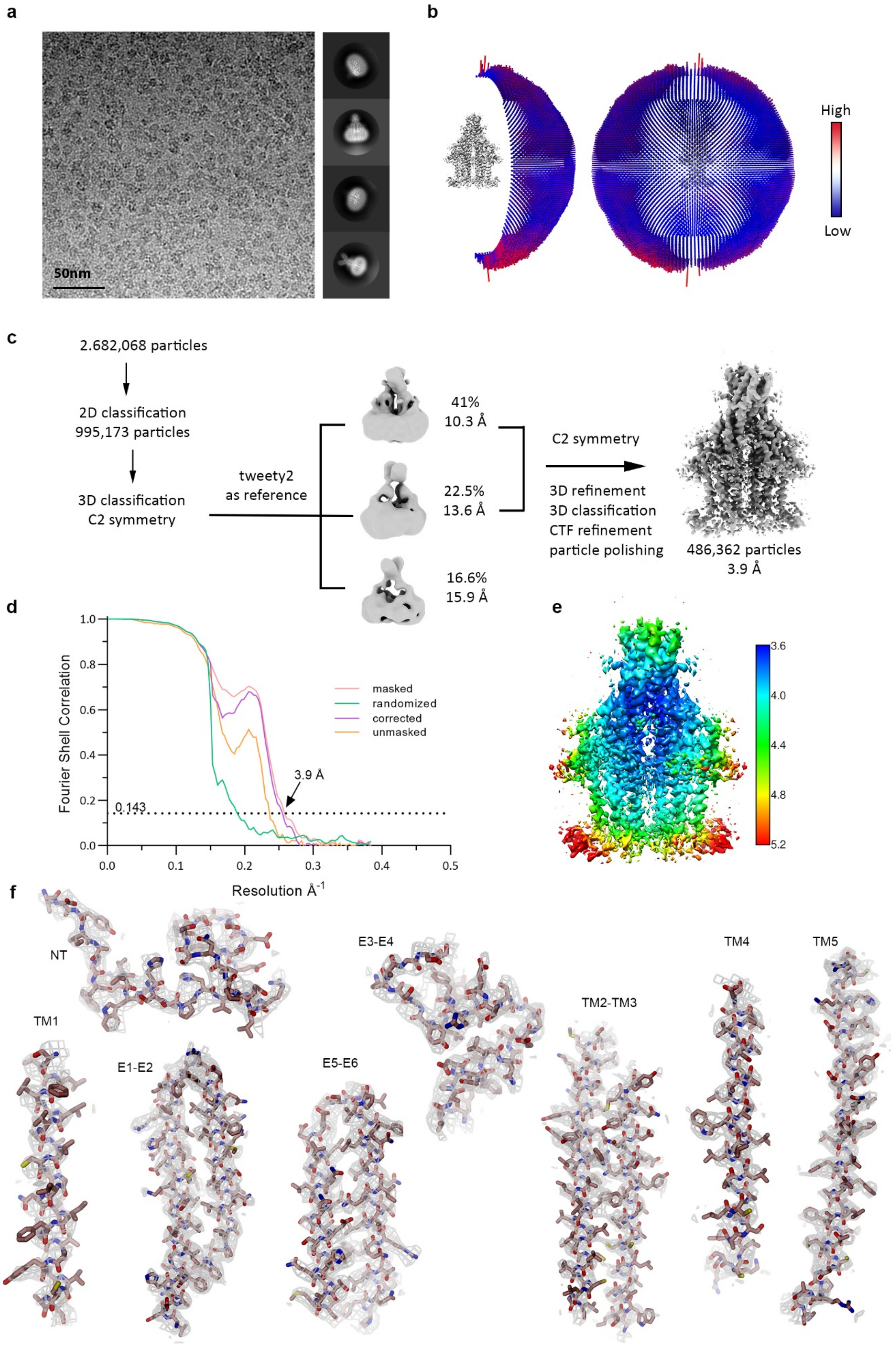
a
50nm
b
High
Low
c
2.682,068 particles
2D classification
995,173 particles
3D classification
C2 symmetry
tweety2
as reference
41%
10.3 Å
22.5%
13.6 Å
16.6%
15.9 Å
C2 symmetry
3D refinement
3D classification
CTF refinement
particle polishing
486,362 particles
3.9 Å
d
Fourier Shell Correlation
Resolution Å-1
masked
randomized
corrected
unmasked
3.9 Å
0.143
1.0
0.8
0.6
0.4
0.2
0.0
0.0
0.1
0.2
0.3
0.4
0.5
e
3.6
4.0
4.4
4.8
5.2
f
NT
E3-E4
TM4
TM5
TM2-TM3
TM1
E1-E2
E5-E6

Supplementary Fig. 3: Cryo-EM structure of TTYH2 in lipid nanodiscs. Representative cryo-EM micrograph (of a total of 6,947) and 2D class averages of TTYH2 in lipid nanodiscs. **b** Angular distribution of particles used for the final 3D reconstruction of TTYH2 in lipid nanodiscs. The length and the color of cylinders correspond to the number of particles with respective Euler angles. **c** Data processing workflow. Particle autopicking was aided by the 3.3 Å TTYH2 map low-pass filtered to 20 Å as a 3D reference and yielded 2,682,068 picked particles, which were subjected to a 2D classification. Subsequent C2-symmetrized 3D classification with the 3.3 Å TTYH2 map as a 3D reference yielded three classes with clear protein features (the distribution of particles and the resolution of each class are indicated). Particles from the 3D classes showing high resolution features were used for map refinement resulting in a 3.9 Å final map. **d** FSC plots of the final TTYH2 reconstruction: unmasked (yellow), masked (pink), randomized (green) and corrected for mask convolution effects (purple). The resolution at which the FSC curve of the map, which was corrected for mask convolution effects drops below 0.143 is indicated. **e** Final 3D reconstruction of TTYH2 in nanodiscs coloured according to local resolution. **f** Sections of the cryo-EM density (grey mesh, contoured at 5.5 σ) superimposed on the model. The different regions of the protein are labelled.

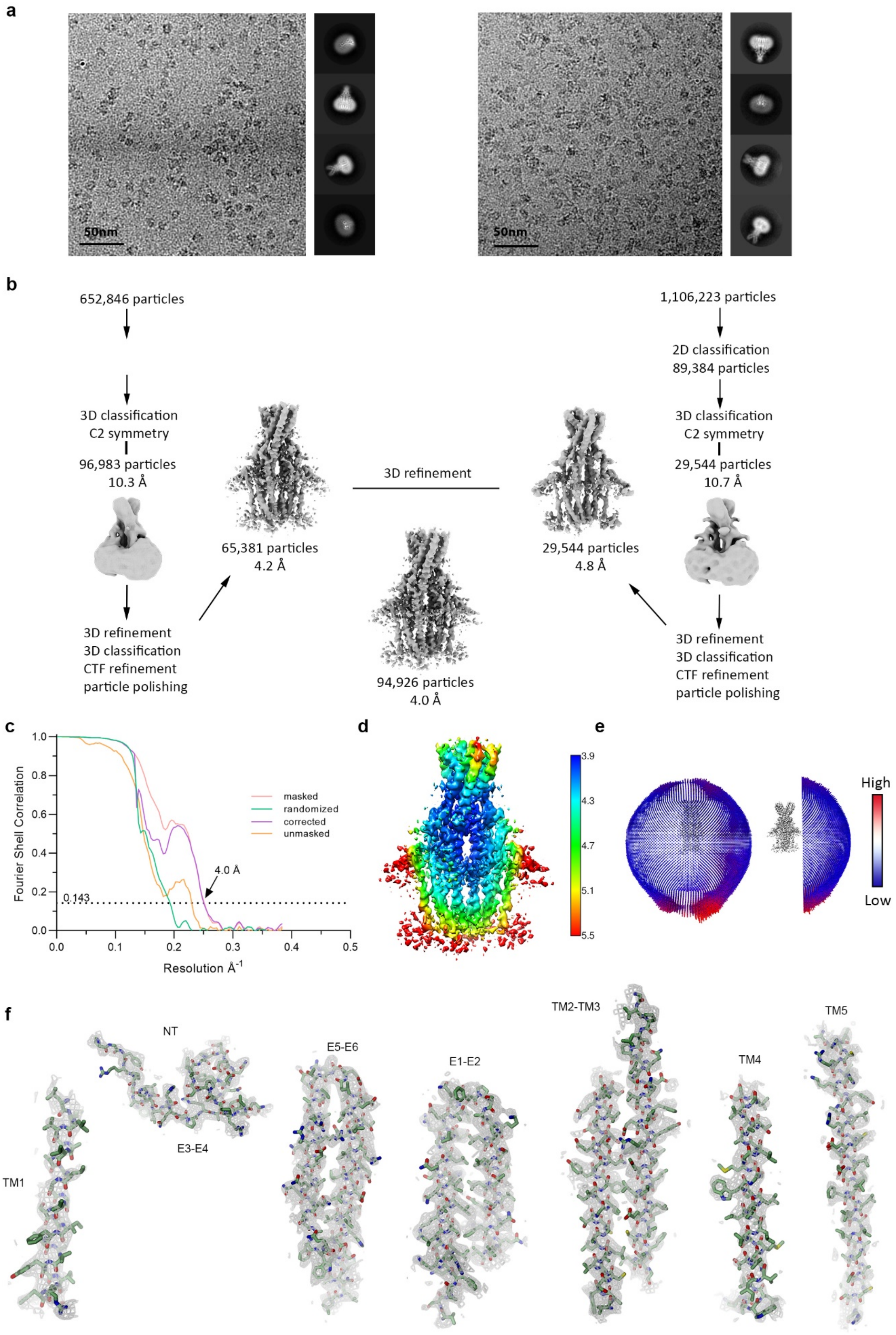
a
50nm
50nm
b
652,846 particles
3D classification
C2 symmetry
96,983 particles
10.3 Å
3D refinement
3D classification
CTF refinement
particle polishing
65,381 particles
4.2 Å
3D refinement
94,926 particles
4.0 Å
29,544 particles
4.8 Å
1,106,223 particles
2D classification
89,384 particles
3D classification
C2 symmetry
29,544 particles
10.7 Å
3D refinement
3D classification
CTF refinement
particle polishing
c
Fourier Shell Correlation
Resolution Å-1
masked
randomized
corrected
unmasked
4.0 Å
0.143
d
3.9
4.3
4.7
5.1
5.5
e
High
Low
f
TM1
NT
E3-E4
E5-E6
E1-E2
TM2-TM3
TM4
TM5

Supplementary Fig. 4: Cryo-EM structure of TTYH1 in GDN. a Representative cryo-EM micrographs (of a total of 5,136 collected for the dataset shown left, and 4,345 for the dataset shown right) and 2D class averages from the two TTYH1 datasets. **b** Data processing workflow. Particle autopicking was aided by the 3.3 Å TTYH2 map low-pass filtered to 20 Å as 3D reference and yielded 652,846 and 1,106,223 picked particles, respectively. Particles from the two individual TTYH1 datasets were processed separately. In each case, the particle set used for map refinement after 2D and 3D classifications had to be further subjected to several rounds of 3D classification without image alignment to discard poorly aligned particles. The two individual TTYH1 maps were refined to 4.2 Å and 4.8 Å, respectively. Particles from both maps were combined and refined further to obtain a final TTYH1 map at 4.0 Å. **c** FSC plots of the final TTYH1 reconstruction: unmasked (yellow), masked (pink), randomized (green) and corrected for mask convolution effects (purple). The resolution at which the FSC curve of the map, which was corrected for mask convolution effects drops below 0.143 is indicated. **d** Final 3D reconstruction of TTYH1 coloured according to local resolution. **e** Angular distribution of particles used for the final 3D reconstruction of TTYH1. The length and the color of cylinders correspond to the number of particles with respective Euler angles. **f** Sections of the cryo-EM density (grey mesh, contoured at 5.5 σ) superimposed on the model. The different regions of the protein are labelled.

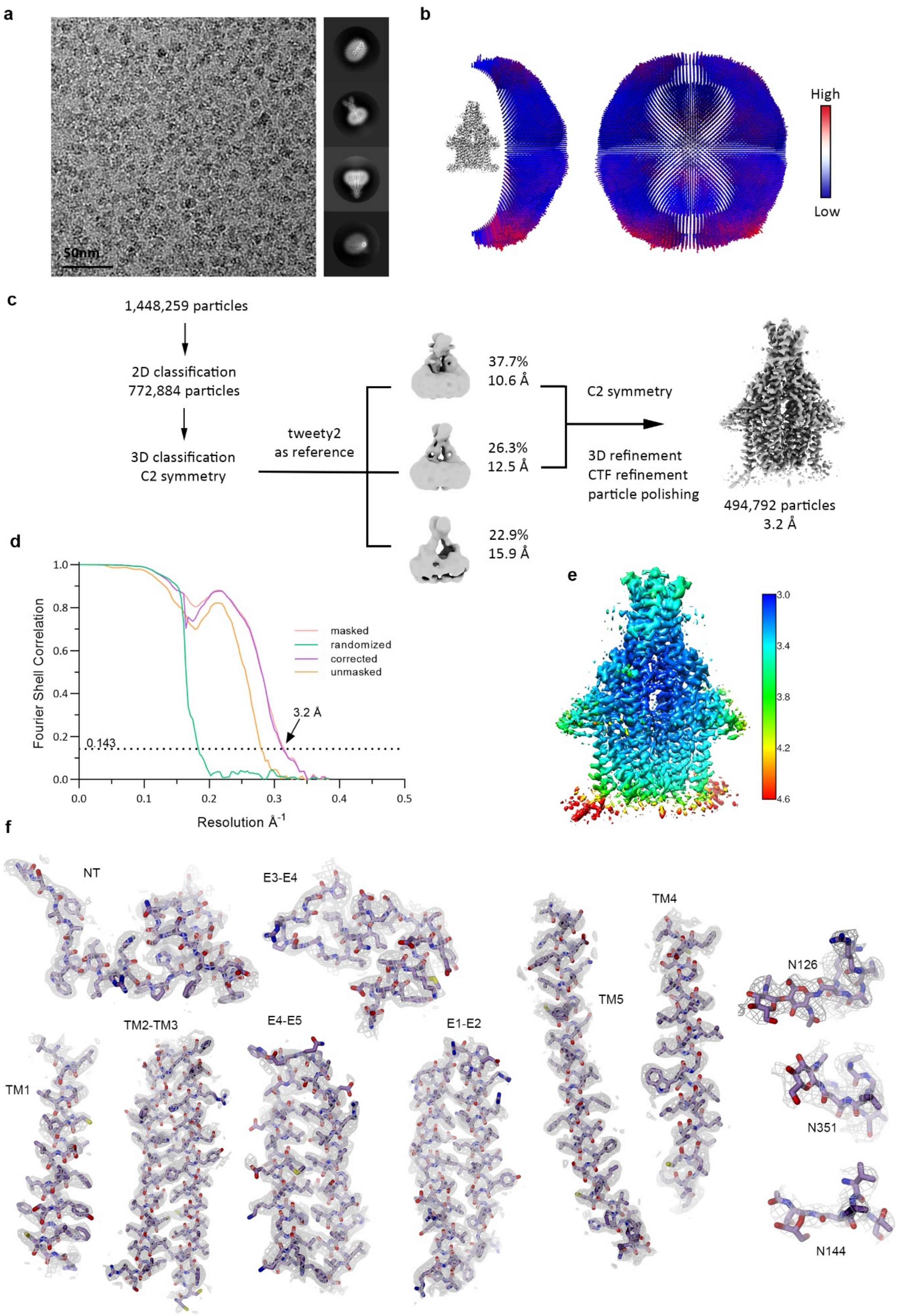

a
50nm
b
High
Low
c
1,448,259 particles
2D classification
772,884 particles
3D classification
C2 symmetry
tweety2
as reference
37.7%
10.6 Å
26.3%
12.5 Å
22.9%
15.9 Å
C2 symmetry
3D refinement
CTF refinement
particle polishing
494,792 particles
3.2 Å
d
Fourier Shell Correlation
Resolution Å-1
masked
randomized
corrected
unmasked
3.2 Å
0.143
e
3.0
3.4
3.8
4.2
4.6
f
NT
E3-E4
TM4
TM5
N126
TM1
TM2-TM3
E4-E5
E1-E2
N351
N144

Supplementary Fig. 5: Cryo-EM structure of TTYH3 in Ca^{2+} and GDN. **a** Representative cryo-EM micrograph (of a total of 3,758) and 2D class averages of TTYH3. **b** Angular distribution of particles used for the final 3D reconstruction of TTYH 3. The length and the color of cylinders correspond to the number of particles with respective Euler angles. **c** Data processing workflow. Particle autopicking was aided by the 3.3 Å TTYH2 map low-pass filtered to 20 Å as 3D reference and yielded 1,448,259 picked particles, which were subjected to a 2D classification. The same 3.3 Å TTYH2 map was used as a 3D reference in the subsequent 3D classification. The distribution of particles and the resolution of best classes are indicated. Particles from 3D classes with high resolution features were refined to a 3.2 Å TTYH3 map. **d** FSC plot of the final TTYH3 reconstruction: unmasked (yellow), masked (pink), randomized (green) and corrected for mask convolution effects (purple). The resolution at which the FSC curve of the map, which was corrected for mask convolution effects drops below 0.143 is indicated. **e** Final 3D reconstruction of TTYH3 coloured according to local resolution. **f** Sections of the cryo-EM density (grey mesh, contoured at 5.5 σ) superimposed on the model. The different regions of the protein are labelled. N126, N144 and N351 correspond to glycosylated residues.

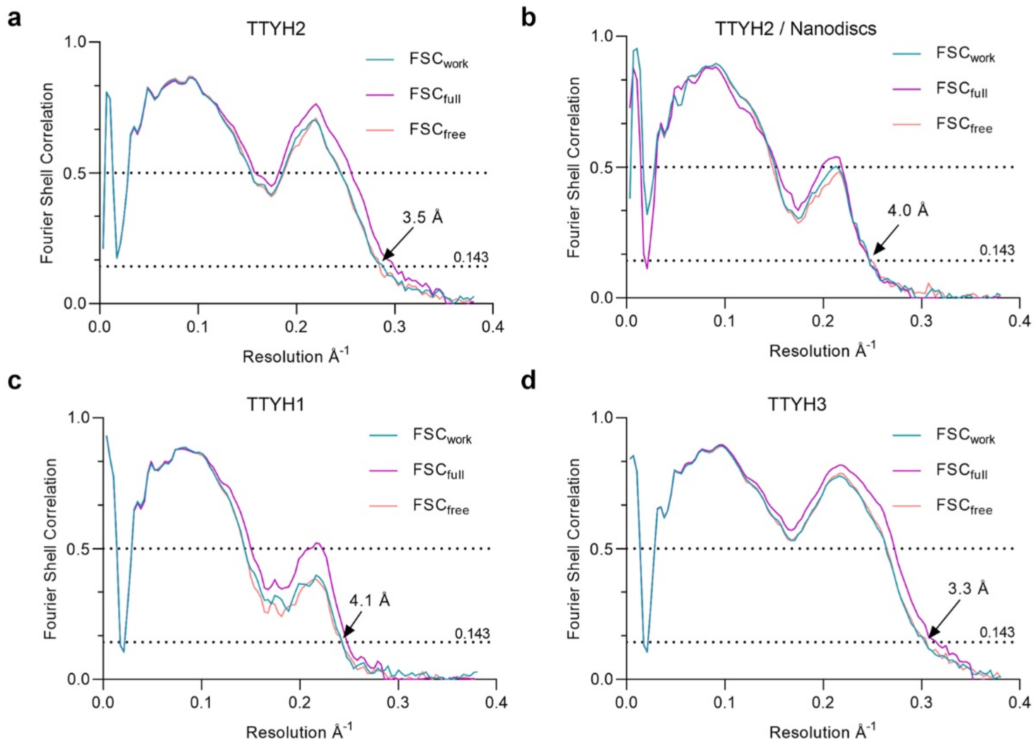

Supplementary Fig. 6: Model validation. FSC plots of atomic TTYH models refined against the respective cryo-EM data. **a** TTYH2 in GDN, **b** TTYH2 in lipid nanodiscs, **c** TTYH1 in GDN and **d** TTYH3 in GDN and Ca^{2+}. FSC_{full} (magenta) is calculated for the full masked map and the model refined against the complete dataset. FSC_{work} (blue) is calculated for the half-map 1 and the model refined against the dataset comprising half map 1. FSC_{free} (orange) is calculated for the half-map 2 and the model refined against the dataset comprising half map 1. The gold-standard FSC threshold (0.143) was used for FSC_{work} and FSC $_{free}$. A FSC threshold of 0.5 was used for FSC_{full}.

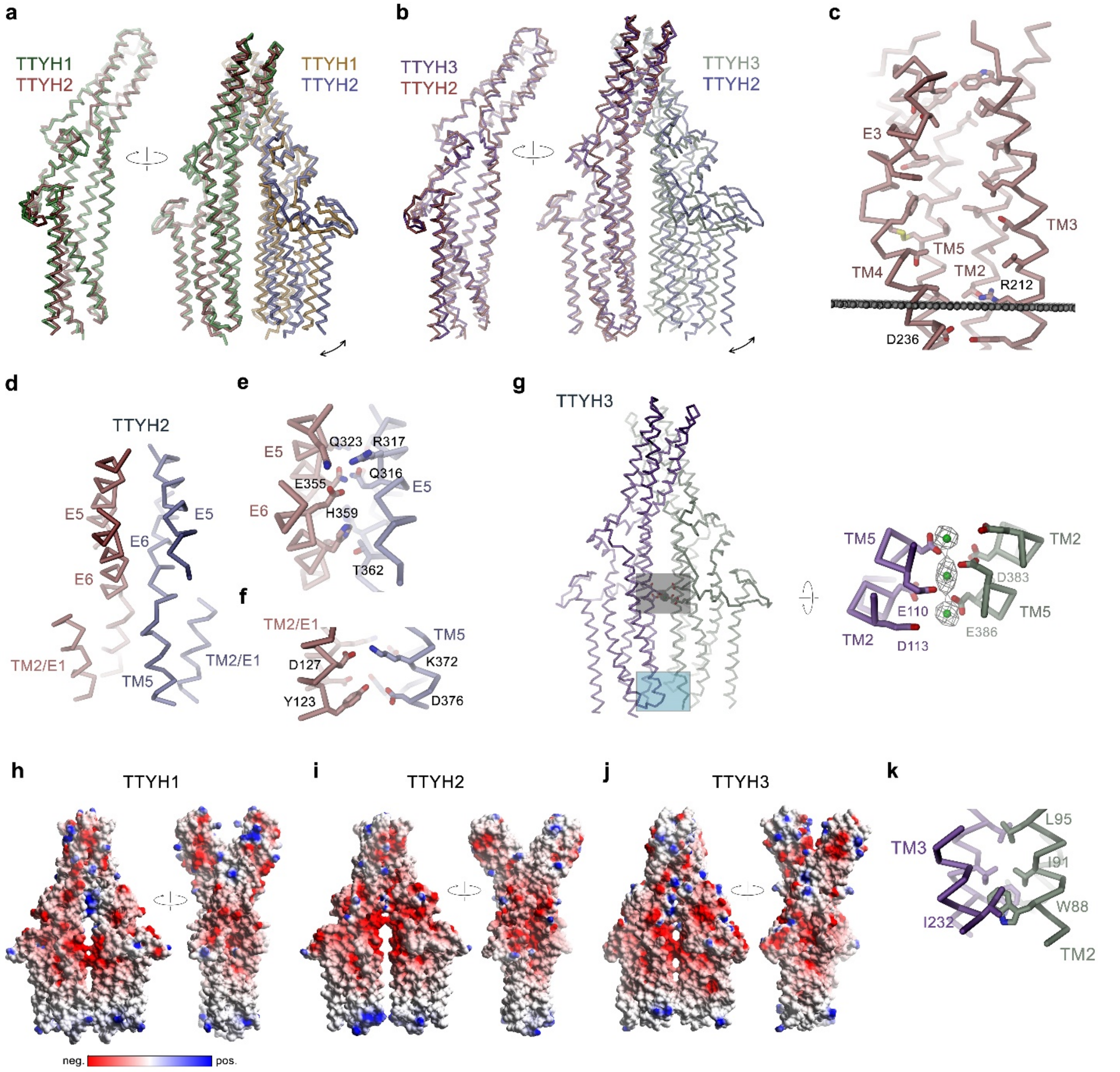

Supplementary Fig. 7: Structural features of TTYH proteins. Superposition of single subunits of TTYH2 and, **a** TTYH1 or, **b** TTYH3. Left, Cα-representation of single superimposed subunits, right, TTYH dimers superimposed on the subunit shown left. Conformational differences in the right subunit due to the altered mutual orientations in the dimeric proteins are indicated by arrows. The relationship between views is indicated. **c** View on the extracellular cavity of TTYH2. The protein is shown as Cα-trace with residues lining the interior of the cavity as sticks. The outer boundary of the hydrophobic membrane core is indicated by grey spheres. **d–f** Dimer interface in the extracellular domain of TTYH2 (**d**). The view is parallel to the membrane. Selected secondary structure elements are labeled. Interacting residues located in the Ex2 (**e**) and the Ex1 region (**f**).

g Presumed binding of Ca^{2+} to an acidic cluster at the dimer interface of TTYH3. The view is from within the membrane, blue rectangle marks transmembrane contact region, grey rectangle marks the region of acidic residues creating a potential Ca^{2+}-binding site. Inset (right) shows a blow-up of the binding site in indicated orientation with cryo-EM density of TTYH3 (grey mesh, contoured at 5.5 σ) shown superimposed on putative Ca^{2+} ions (green). **h–j** Electrostatic potential of TTYH1 (**h**), TTYH2 (**i**) and TTYH3 (**j**) mapped on their molecular surface. Blue, positive, red, negative potential. **k** Inter-subunit contacts at the intracellular end of the TMD (see blue rectangle in **g** for orientation). **a–g, k** The protein is shown as Cα-trace with selected residues as sticks.

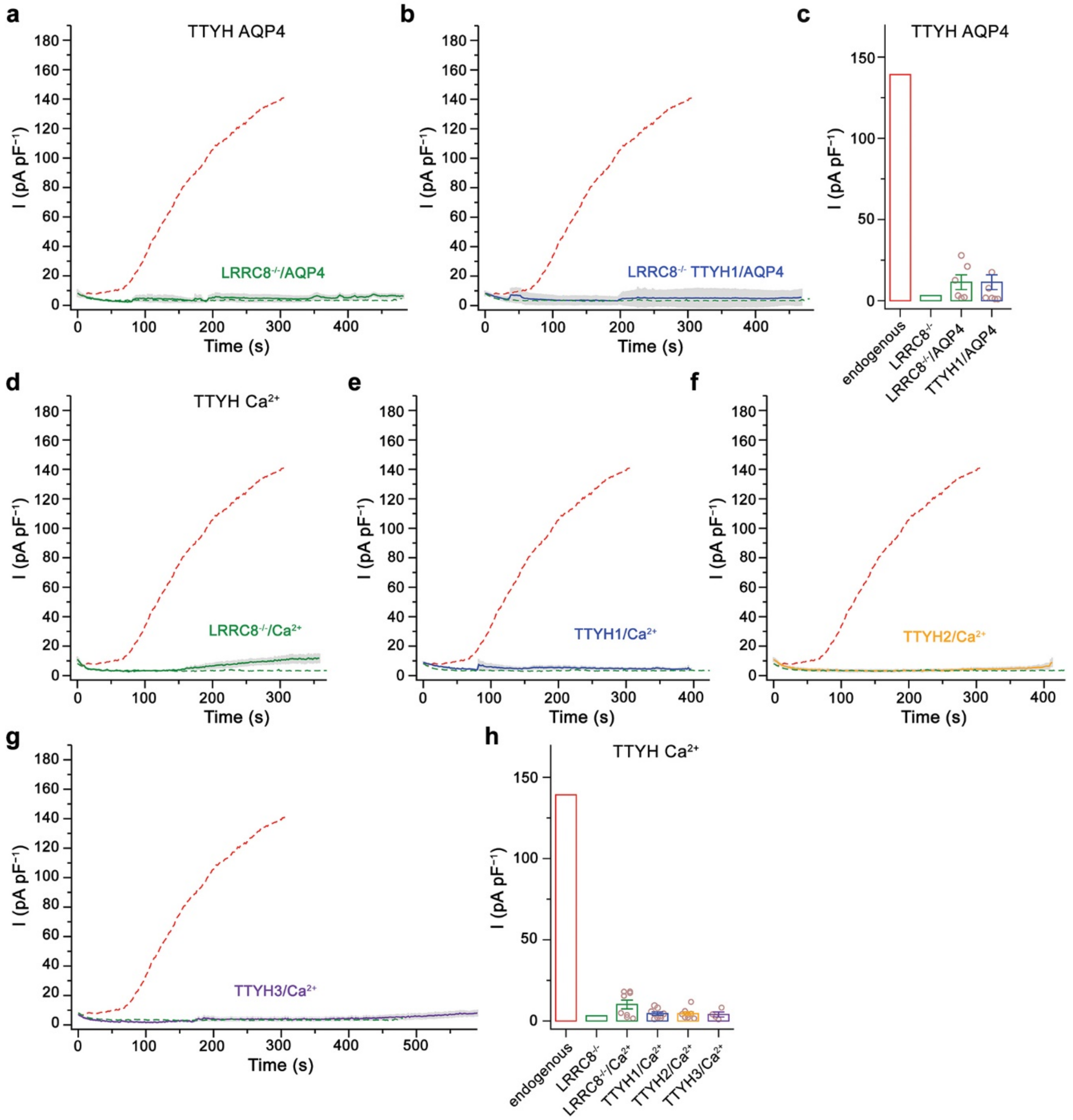

Supplementary Fig. 8: Electrophysiology 1. Response of cells exposed to different conditions. Currents are measured by patch-clamp in the whole-cell configuration at 100 mV. **a, b** Response of cells overexpressing AQP4 to hypotonic conditions (starting at t=0 and lasting for the entire duration of the recording). Average current density of LRRC8$^{-/-}$ cells transfected with **a** AQP4 (n=6), and **b** AQP4 and TTYH1 (n=6). **c** Current response (at 100 mV) of cells expressing the indicated constructs recorded 300 seconds after exposure to hypotonic medium (endogenous, n=9; LRRC8$^{-/-}$, n=9; LRRC8$^{-/-}$/AQP4, n= 6; TTYH1/AQP4, n=6). **d–g** Current response at 1mM free Ca^{2+} in the pipette solution. Average current density of **d** LRRC8$^{-/-}$ cells (n=8) and LRRC8$^{-/-}$ cells transfected with **e** TTYH1 (n=9), **f** TTYH2 (n=8) and **g** TTYH3 (n=4). **h** Current response (at 100 mV) of cells expressing the indicated constructs recorded 300 seconds after exposure to 1mM of free Ca^{2+} (endogenous, n=9; LRRC8$^{-/-}$, n=9; LRRC8$^{-/-}$/Ca^{2+}, n=8; TTYH1/Ca^{2+}, n=9, TTYH2/Ca^{2+}, n=8; TTYH3/Ca^{2+}, n=4). **a**, **b, d–g** Mean values of endogenous currents of HEK293 cells (red) and LRRC8$^{-/-}$ cells (green) shown in Fig. 4a are displayed as dashed line for comparison. **c**, **h** Values from individual measurements are shown as circles, mean values as bars. Endogenous and LRRC8$^{-/-}$ refer to mean values shown in Fig. 4f and are displayed as reference. Differences between values obtained from LRRC8$^{-/-}$ cells and TTYH-expressing under indicated conditions cells were analyzed in a two-sample two-sided t-test and found to be non-significant (**c** comparison between LRRC8$^{-/-}$/AQP4 and TTYH1/AQP4 p=0.19, **h** comparison between LRRC8$^{-/-}$ and TTYH constructs, all in presence of Ca^{2+}: TTYH1 p=0.08, TTYH2 p=0.08, TTYH3 p=0.07).

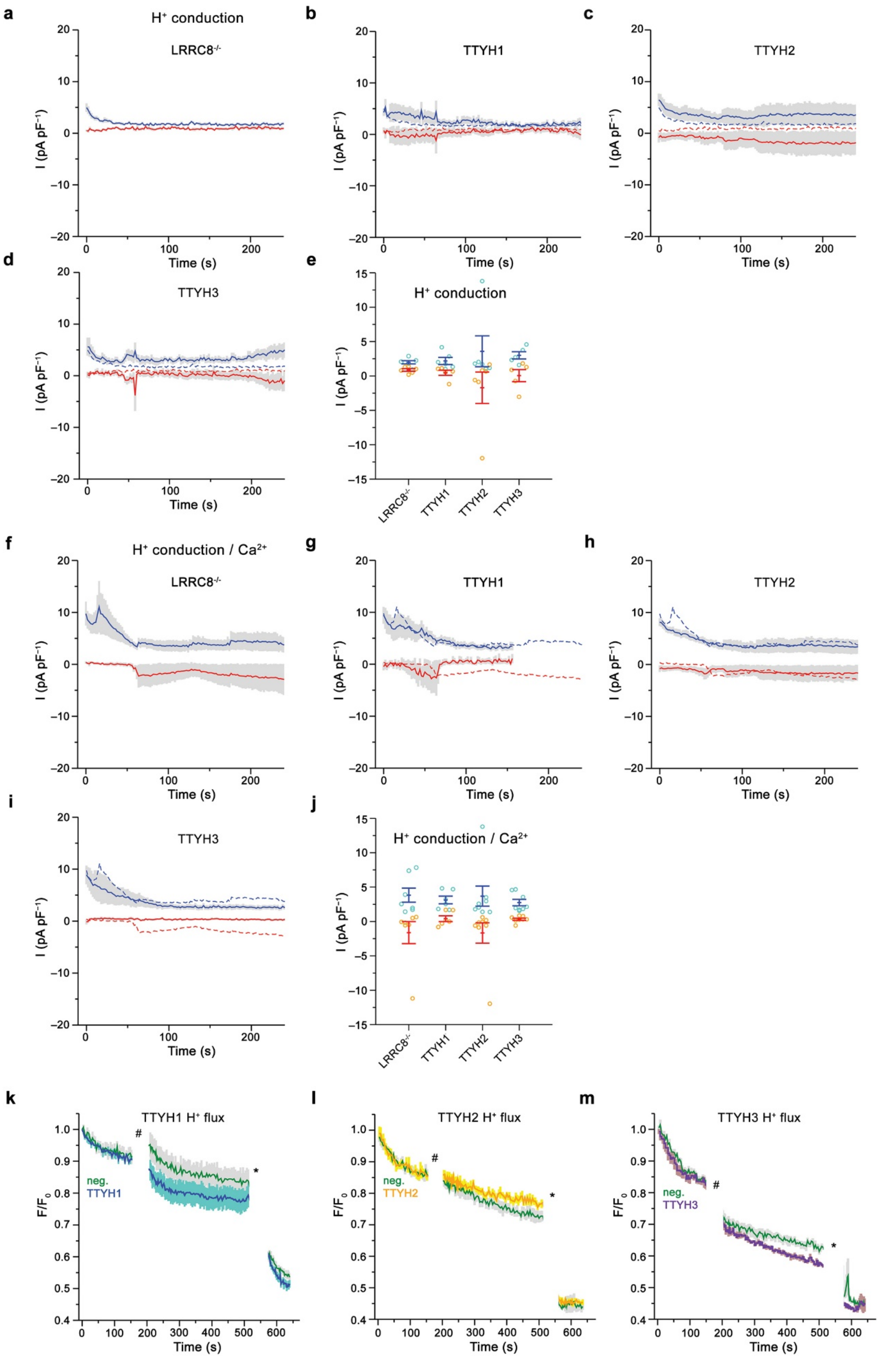
a
H⁺ conduction
LRRC8-/-
b
TTYH1
c
TTYH2
d
TTYH3
e
H⁺ conduction
I (pA pF⁻¹)
Time (s)
LRRC8-/-
TTYH1
TTYH2
TTYH3
f
H⁺ conduction / Ca²⁺
LRRC8-/-
g
TTYH1
h
TTYH2
i
TTYH3
j
H⁺ conduction / Ca²⁺
k
TTYH1 H⁺ flux
neg.
TTYH1
l
TTYH2 H⁺ flux
neg.
TTYH2
m
TTYH3 H⁺ flux
neg.
TTYH3
F/F0
#
*

Supplementary Fig. 9: Electrophysiology 2 and ion transport assays. Response of cells exposed to low extracellular pH. Currents are measured by patch-clamp in the whole-cell configuration. **a–j** H^+ conduction. H^+ current response (100 mV, blue and –100mV, red) of cells expressing the indicated constructs recorded 300 seconds after exposure to hypotonic medium. **a–d** H^+ current response in absence of Ca^{2+}. Average current density of **a** LRRC8$^{–/–}$ cells (n=7), and LRRC8$^{–/–}$ cells transfected with **b** TTYH1 (n=6), **c** TTYH2 (n=6) and **d** TTYH3 (n=5). **e** H^+ current response (at 100 mV and –100mV) of cells expressing the indicated constructs recorded 150 seconds after exposure to hypotonic medium (LRRC8$^{-/-}$, n = 7; TTYH1, n = 6; TTYH2, n = 6; TTYH3, n = 5). **f–i** H^+ current response in presence of 1 mM Ca^{2+} in the pipette solution. Average current density of **f** LRRC8$^{–/–}$ cells (n=7), and LRRC8$^{–/–}$ cells transfected with **g** TTYH1 (n=6), **h** TTYH2 (n=8) and **i** TTYH3 (n=8). **j** H^+ current response (at 100 mV and –100mV) of cells expressing the indicated constructs recorded 150 seconds after exposure to hypotonic medium in presence of 1mM Ca^{2+} in the pipette solution (LRRC8$^{-/-}$, n = 7; TTYH1, n = 6; TTYH2, n = 7; TTYH3, n = 8). **b–d**, **g–i** Mean values of endogenous currents of LRRC8$^{–/–}$ cells shown in **a** or **f** are displayed as dashed line for comparison. **e**, **j** Values from individual measurements are shown as circles (100 mV cyan, –100 mV orange), mean values and errors as bars. Differences between values obtained from LRRC8$^{–/–}$ cells and TTYH-expressing cells were analyzed in a two-sample two-sided t-test and found to be non-significant (**e** H^+ currents in absence of Ca^{2+}: TTYH1 100 mV p=0.73, –100 mV p=0.33; TTYH2 100 mV p=0.47, –100 mV p=0.27; TTYH3 100 mV p=0.14, –100 mV p=0.42. **j** H^+ currents in presence of Ca^{2+}: TTYH1 100 mV p=0.57, –100 mV p=0.26; TTYH2 100 mV p=0.94, –100 mV p=0.98; TTYH3 100 mV p=0.37, –100 mV p=0.27). **k–m** H^+ flux into proteoliposomes containing reconstituted TTYH proteins. Fluorescence quenching of the pH sensitive fluorophore ACMA upon H^+ influx driven by a negative membrane potential established upon addition of the K^+ ionophore valinomycin (#). * indicates the addition of the protonophore CCCP. Proteoliposomes contain, **k** TTYH1, **l** TTYH2 and, **m** TTYH3. Data from mock reconstituted liposomes generated from the same batch of destabilized lipids are shown as control (neg.). **k–m** Data show mean of four technical replicates. **a–m** errors are s.e.m.

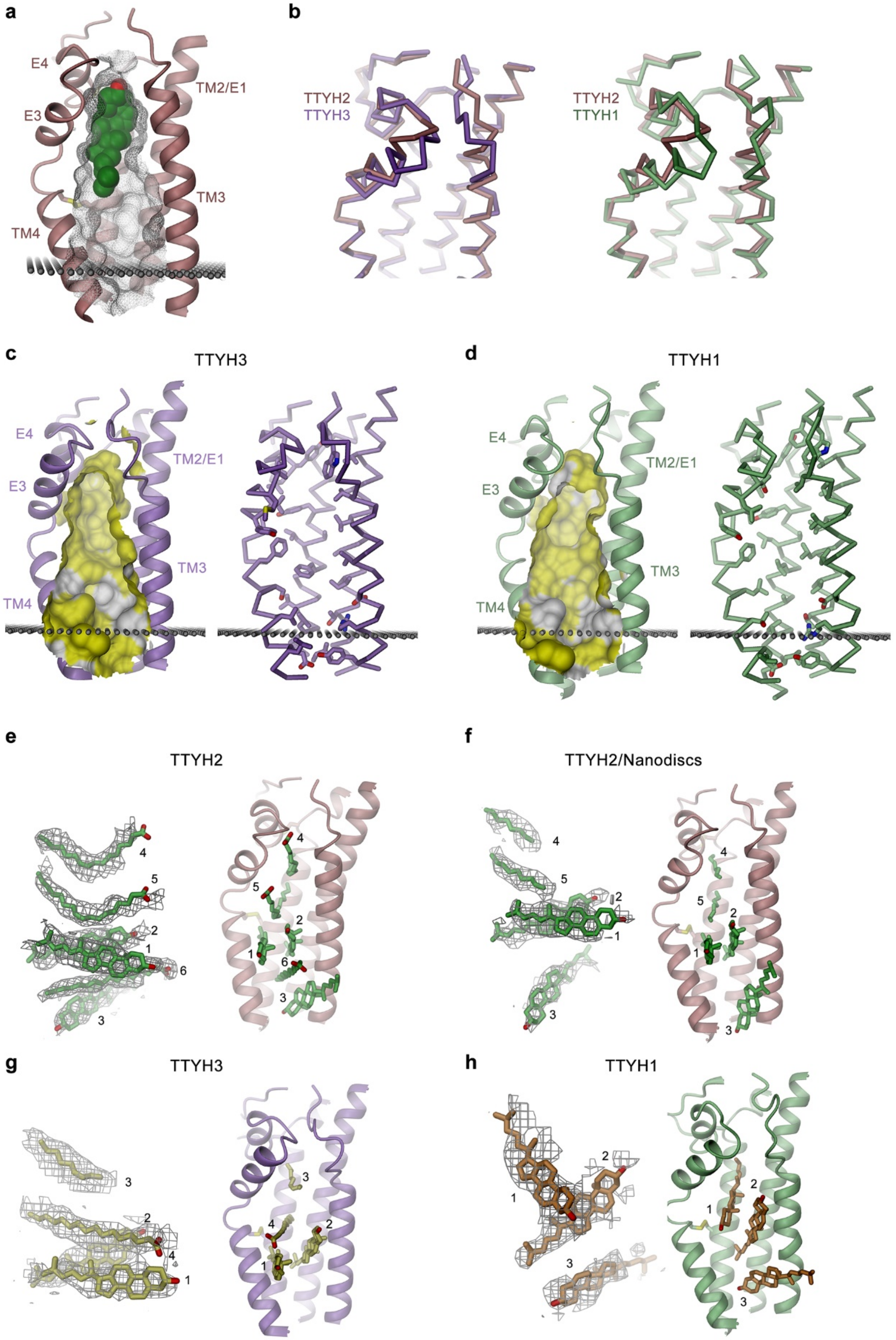

a
b
E4
E3
TM2/E1
TM3
TM4
TTYH2
TTYH3
TTYH2
TTYH1
c
TTYH3
d
TTYH1
e
TTYH2
f
TTYH2/Nanodiscs
g
TTYH3
h
TTYH1

Supplementary Fig. 10: Lipid distribution in the extracellular cavity. a View of the extracellular cavity of TTYH2. The protein is represented as ribbon, the molecular surface of the cavity as mesh. A cholesterol molecule (green CPK model) was modeled to illustrate the size and geometry of the binding site. Grey spheres indicate the outer boundary of the hydrophobic membrane core. **b** Close-up of the region of the extracellular cavity of a superposition of subunits of TTYH2 on TTYH3 (left) and TTYH1 (right). The proteins are shown as Cα-traces. **c**, **d** Extracellular cavity of TTYH3 (**c**) and TTYH1 (**d**). The view is as in (**a**). Left, ribbon representation of the protein with molecular surface displayed. Regions contacted by hydrophobic and aromatic residues are colored in yellow. Right, Cα-representation with residues lining the interior of the cavity shown as sticks. **e–h** Distribution of residual density in the extracellular cavity of TTYH2 (**e**), TTYH2 in nanodiscs (**f**), TTYH3 (**g**) and TTYH1 (**h**). Left, Cryo-EM density (contoured at 5.5 σ, grey mesh) arbitrarily interpreted as either cholesterol or an acyl chain for size comparison. The view of the protein is as in Fig. 5a. Right, extracellular cavity with modeled lipid molecules. The view is as in **a**. Numbers indicate the corresponding positions.

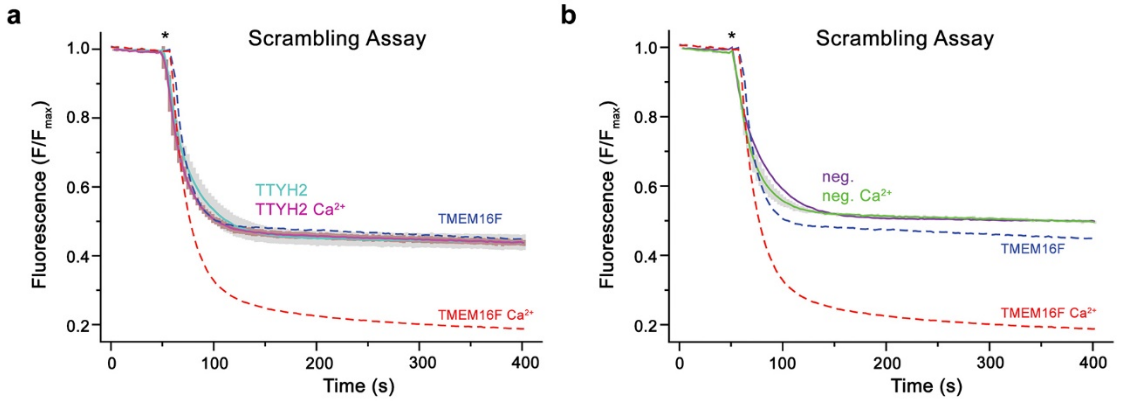

Supplementary Fig. 11: Lipid scrambling assay. Assay of the protein-mediated movement of fluorescent lipids between both leaflets of a bilayer. The addition of the reducing agent dithionite to the solution (*) bleaches the fluorescent groups located in the outer leaflet of the membrane, which leads to a decrease of the initial fluorescence to about half (45-55% depending on the preparation) in absence of scrambling activity and a further reduction in case of lipid flip-flop. **a** Data show traces from proteoliposomes containing TTYH2 in absence and presence of 2 mM Ca^{2+}. **b** Traces of liposomes not containing any protein in presence and absence of 2 mM Ca^{2+}. **a**, **b** Data show mean and standard deviations of indicated technical replicates (neg., n=2; neg. Ca^{2+}, n=3; TTYH2, n=5; TTYH2 Ca^{2+}, n=3) errors are s.d.. Traces of TMEM16F obtained from a previous study[36] in absence and presence of 100 µM Ca^{2+} are shown as reference for an inactive and an active scramblase, respectively (dashed lines).

a b

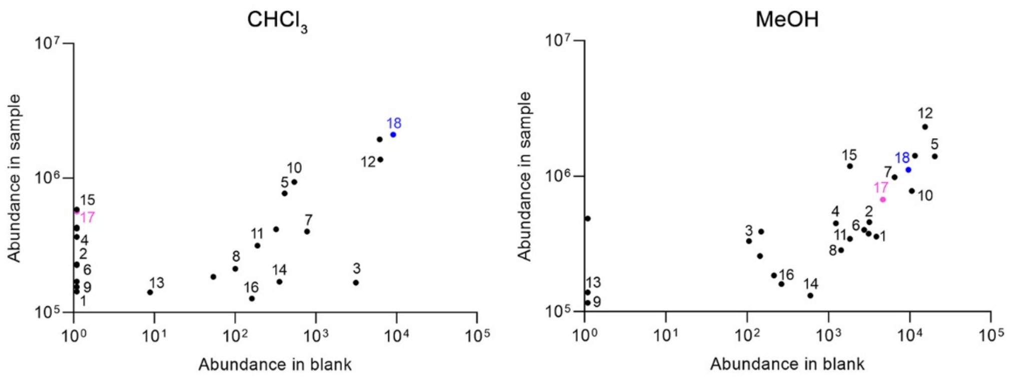

c

compound	compound class	retention time (min)	m/z	mass error (ppm)	isotope similarity (%)
1	glycerophospholipid	10.01	480.34	-0.25	92.9
2	glycerophospholipid	10.11	438.3	0.43	95.7
3	glycerophospholipid	10.18	464.31	0.57	95.5
4	glycerophospholipid	10.3	634.41	0.48	92.7
5	glycerophospholipid	10.35	481.31	0.7	97.2
6	glycerophospholipid	10.38	550.39	0.26	95.3
7	glycerophospholipid	10.48	703.48	0.63	93.6
8	glycerophospholipid	10.61	465.32	0.57	95
9	glycerophospholipid	11.52	776.58	0.19	95.3
10	glycerophospholipid	11.72	677.5	0.057	97.4
11	glycerophospholipid	12.49	744.55	0.5	96.7
12	glycerophospholipid	12.49	785.59	0.18	98.5
13	glycerophospholipid	12.62	702.54	0.16	97
14	glycerophospholipid	12.71	746.57	0.21	97
15	glycerophospholipid	12.71	787.61	0.22	98
16	glycerophospholipid	9.84	714.43	0.1	94.5
17	sphingolipid	11.99	702.57	0.34	97
18	prostaglandin	8.32	352.23	0.3	96.5

Supplementary Fig. 12: Lipid analysis. LC-MS analysis of extracts of a protein preparation of TTYH2 purified as for structural analysis. **a**, **b** Scatter plot depicting the relative abundance of compounds in comparison to a blank consisting of the purification buffer. **a** Chloroform, **b** methanol extracts. Numbers indicate identified corresponding molecules. **c** Table summarizing the properties of compounds shown in **a** and **b**. The chemical identity was identified in a search against the LipidMaps (LM) library with matching tolerances of 1 ppm in mass accuracy and >90% in isotope similarity.
