# Supplementary material for: Cryo-EM structures of the TTYH family reveal a novel architecture for lipid interactions
Source: Nat Commun. 2021 Aug 12;12:4893. doi: 10.1038/s41467-021-25106-4 (PMC8361169; doi:10.1038/s41467-021-25106-4)
Supplement: Supplementary file 3 — Reporting Summary [file 41467_2021_25106_MOESM3_ESM.pdf]

nature research

Corresponding author(s): Raimund Dutzler
Marta Sawicka

Last updated by author(s): Jul 16, 2021

Reporting Summary

Nature Research wishes to improve the reproducibility of the work that we publish. This form provides structure for consistency and transparency in reporting. For further information on Nature Research policies, see our Editorial Policies and the Editorial Policy Checklist.

Statistics

For all statistical analyses, confirm that the following items are present in the figure legend, table legend, main text, or Methods section.

n/a	Confirmed	
☐	☒	The exact sample size (*n*) for each experimental group/condition, given as a discrete number and unit of measurement
☐	☒	A statement on whether measurements were taken from distinct samples or whether the same sample was measured repeatedly
☐	☒	The statistical test(s) used AND whether they are one- or two-sided<br>*Only common tests should be described solely by name; describe more complex techniques in the Methods section.*
☐	☒	A description of all covariates tested
☐	☒	A description of any assumptions or corrections, such as tests of normality and adjustment for multiple comparisons
☐	☒	A full description of the statistical parameters including central tendency (e.g. means) or other basic estimates (e.g. regression coefficient) AND variation (e.g. standard deviation) or associated estimates of uncertainty (e.g. confidence intervals)
☐	☒	For null hypothesis testing, the test statistic (e.g. F, t, r) with confidence intervals, effect sizes, degrees of freedom and P value noted<br>*Give P values as exact values whenever suitable.*
☒	☐	For Bayesian analysis, information on the choice of priors and Markov chain Monte Carlo settings
☒	☐	For hierarchical and complex designs, identification of the appropriate level for tests and full reporting of outcomes
☒	☐	Estimates of effect sizes (e.g. Cohen's *d*, Pearson's *r*), indicating how they were calculated

Our web collection on statistics for biologists contains articles on many of the points above.

Software and code

Policy information about availability of computer code

Data collection	Clampex 10.7, EPU 2.7.
Data analysis	Clampfit 10.7, Excel 2019, MotionCorr2 1.1.0, CTFFIND 4.1, Relion 3.0.9, Relion 3.1, Phenix 1.18, Coot 0.8.9.2, DINO 0.9.6, ChimeraX 1.2.5, Chimera 1.15.

For manuscripts utilizing custom algorithms or software that are central to the research but not yet described in published literature, software must be made available to editors and reviewers. We strongly encourage code deposition in a community repository (e.g. GitHub). See the Nature Research guidelines for submitting code & software for further information.

Data

Policy information about availability of data

All manuscripts must include a data availability statement. This statement should provide the following information, where applicable:

- Accession codes, unique identifiers, or web links for publicly available datasets
- A list of figures that have associated raw data
- A description of any restrictions on data availability

Cryo-EM maps have been deposited in the Electron Microscopy Data Bank under accession numbers EMD-13194, EMD-13201, EMD-13200 and EMD-13198. Coordinates for the models of the full-length TTYH2, TTYH2 in nanodiscs, TTYH1 and TTYH3 have been deposited in the Protein Data Bank under accession numbers 7P54, 7P5M, 75PJ, and 7P5C, respectively. The data from electrophysiological recordings and the liposome assay showing the absence of chloride and proton conduction in TTYH proteins have been deposited in the Dryad database (DOI 10.5061/dryad.fn2z34ttx), and included in the Source Data file. The data from the scrambling assay and lipidomics of TTYH2 have been deposited in the Dryad database (DOI 10.5061/dryad.69p8cz92n), and included in the Source Data file. Data supporting the findings of this study are available from the corresponding authors upon reasonable request.

Field-specific reporting

Please select the one below that is the best fit for your research. If you are not sure, read the appropriate sections before making your selection.

- [x] Life sciences
- [ ] Behavioural & social sciences
- [ ] Ecological, evolutionary & environmental sciences

For a reference copy of the document with all sections, see nature.com/documents/nr-reporting-summary-flat.pdf

Life sciences study design

All studies must disclose on these points even when the disclosure is negative.

Sample size	No sample size determination was performed. Functional experiments were performed multiple times with similar results and further inclusion of data did not change the results. Complete Cryo-EM statistics are provided in Table 1, and supplementary figures 2-5.
Data exclusions	Data selection for Cryo-EM is illustrated in Table 1 and supplementary figures 2-5. In electrophysiological experiments, leaky recordings were discarded. Otherwise no data were excluded from the analyses.
Replication	Electrophysiology data show the mean of the indicated number of biological replicates and errors are indicated. Recordings were performed multiple times from different transfections and all replications were successful. Liposome-based assays were performed in several technical replicates and all replications were successful.
Randomization	Randomization is not relevant for this study, as there were no groups allocated in any of the experiments
Blinding	Not applicable as this is deemed not practically feasible.

Reporting for specific materials, systems and methods

We require information from authors about some types of materials, experimental systems and methods used in many studies. Here, indicate whether each material, system or method listed is relevant to your study. If you are not sure if a list item applies to your research, read the appropriate section before selecting a response.

Materials & experimental systems

n/a	Involved in the study	
☐	☒	Antibodies
☐	☒	Eukaryotic cell lines
☒	☐	Palaeontology and archaeology
☒	☐	Animals and other organisms
☒	☐	Human research participants
☒	☐	Clinical data
☒	☐	Dual use research of concern

Methods

n/a	Involved in the study	
☒	☐	ChIP-seq
☒	☐	Flow cytometry
☒	☐	MRI-based neuroimaging

Antibodies

Antibodies used	The antibodies used in this study are commercially available and include a mouse anti-c-Myc primary antibody (Sigma, M4439, clone 9E10), and a peroxidase-conjugated goat anti-mouse secondary antibody (Jackson ImmunoResearch, 115-035-146).
Validation	The mouse anti-c-Myc primary antibody was validated by the supplier and the validation report is available from the supplier website: https://www.sigmaaldrich.com/CH/de/product/sigma/m4439?gclid=CjwKCAjwlrqHBhByEiwAnLmYUFiJlYrbnKNzWOVcPLGCKz_dcNwZ-HPPIW3hTaLp1VymEYqu4a-TnxoCVn4QAvD_BwE.

Eukaryotic cell lines

Policy information about cell lines

Cell line source(s)	HEK293T (ATCC, CLR-1573), HEK293S GnTI– (ATCC, CLR-3022).
Authentication	No further authentication was performed for commercially available cell lines. The LRRC8-/- HEK 293 cell line was obtained from the laboratory of T.J. Jentsch. The lack of expression of LRRC8 proteins in latter was confirmed by electrophysiology and Western blots.
Mycoplasma contamination	The cell lines were tested and are free from mycoplasma contamination.

Commonly misidentified lines<br>(See ICLAC register)	No commonly misidentified cell lines were used in this study.
